# Comparative study between cortical bone graft versus bone dust for reconstruction of cranial burr holes

**DOI:** 10.4103/2152-7806.74160

**Published:** 2010-12-22

**Authors:** Paulo V. Worm, Nelson P. Ferreira, Mario B. Faria, Marcelo P. Ferreira, Jorge L. Kraemer, Marcus V. M. Collares

**Affiliations:** Department of Neurosurgery, Hospital São José, Complexo Hospitalar Santa Casa, Brazil; 1 Department of Plastic Surgery, Hospital de Clínicas de Porto Alegre, UFRGS, Porto Alegre, RS, Brazil

**Keywords:** Autograft, bone dust, cranioplasty, surgery

## Abstract

**Background:**

As a consequence of the progressive evolution of neurosurgical techniques, there has been increasing concern with the esthetic aspects of burr holes. Therefore, the objective of this study was to compare the use of cortical bone graft and bone dust for correcting cranial deformities caused by neurosurgical trephines.

**Methods:**

Twenty-three patients were enrolled for cranial burr hole reconstruction with a 1-year follow-up. A total of 108 burr holes were treated; 36 burr holes were reconstructed with autogenous cortical bone discs (33.3%), and the remaining 72 with autogenous wet bone powder (66.6%). A trephine was specifically designed to produce this coin-shaped bone plug of 14 mm in diameter, which fit perfectly over the burr holes. The reconstructions were studied 12 months after the surgical procedure, using three-dimensional quantitative computed tomography. Additionally, general and plastic surgeons blinded for the study evaluated the cosmetic results of those areas, attributing scores from 0 to 10.

**Results:**

The mean bone densities were 987.95 ± 186.83 Hounsfield units (HU) for bone fragment and 473.55 ± 220.34 HU for bone dust (*P* < 0.001); the mean cosmetic scores were 9.5 for bone fragment and 5.7 for bone dust (*P* < 0.001).

**Conclusions:**

The use of autologous bone discs showed better results than bone dust for the reconstruction of cranial burr holes because of their lower degree of bone resorption and, consequently, better cosmetic results. The lack of donor site morbidity associated with procedural low cost qualifies the cortical autograft as the first choice for correcting cranial defects created by neurosurgical trephines.

## INTRODUCTION

Cranioplasty for correcting defects produced by surgical trephines is one of the neurosurgical problems that remain without a proper solution. The large number of alloplastic implants available to perform this procedure corroborates this statement.[3,8,9,15,18,19] The progressive evolution of neurosurgical techniques has provided favorable outcomes to most patients, leading to increasing concerns regarding the esthetic aspects related to the treatment. In this scenario, dissatisfaction with the cosmetic results of cranioplasties motivated this study. The objective of the present study was to compare the widespread use of bone powder collected from skull drilling and a new method, an autogenous cortical bone graft from the inner table of the cranial vault, for filling in the defects produced by neurosurgical trephines in patients undergoing surgical treatment.

## MATERIALS AND METHODS

A prospective study was conducted. Patients undergoing neurosurgical treatment had their burr holes reconstructed using bone dust or cortical bone discs. Three-dimensional quantitative computed tomography (QCT) was performed 12 months after surgery to measure the bone densities of the burr holes. In addition, one general and one plastic surgeon blinded for the study evaluated the cosmetic results of these areas, attributing scores from 0 to 10 (poor to adequate cosmetic result, respectively). Statistical analysis was done using the Statistical Package for the Social Sciences (SPSS) for Windows. Comparison between groups was performed with Student’s t test, with a level of significance of 95% (*P* < 0.05). Approval from the local ethics committee was obtained.

### Surgical procedure

**Figure 1:**
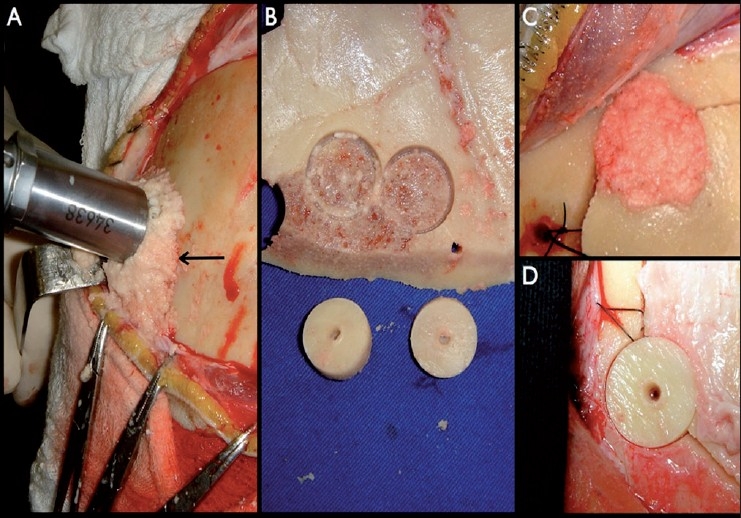
Bone dust (arrow) collected during cranial trepanation (A) and cortical bone discs created from the inner table of the craniotomy flap (B) were used to correct the defect produced by the trephine (C and D, respectively)

An electrical trephine was used for cranial drilling under continuous irrigation with saline in order to avoid heat production and to keep the bone dust wet and easily collectable. The burr holes measured 14 mm in diameter and were bound together with a gigli saw to create the desired bone flap. Wet bone powder was collected with a spoon after each burr hole was drilled and stored in a solution containing 100 mL of saline and 80 mg of gentamicin. For closure, this bone paste was used to completely fill in the burr holes. Alternatively, from the inner surface of the bone flap, which was elevated to perform the intracranial procedure, a 2- to 3-mm thick coin-shaped bone disc matching the burr hole’s diameter was obtained using a trephine. The procedure consisted of drilling the bone flap’s inner table until reaching the diploe. Then, using an osteotome, this circular fragment of cortical bone was separated from the outer cortical table and placed over the burr hole as a bone plug with perfect fit [Figure 1].

### Three-dimensional computed tomography of the skull

Tomographic images of the skull were obtained 8 months after surgery with a 16-channel multi-slice scanner, performing 0.6- or 1.2-mm thick slices in helical mode (GE HiSpeed Advantage Scanner, GE Medical Systems Inc., Milwaukee, WI, USA). Bone density in the reconstructed burr hole area was measured using a tomographic software (Advantage Windows Workstation 4.2, GE Medical Systems Inc., Milwaukee, WI, USA). Results were presented in Hounsfield units (HU).

## RESULTS

**Figure 2:**
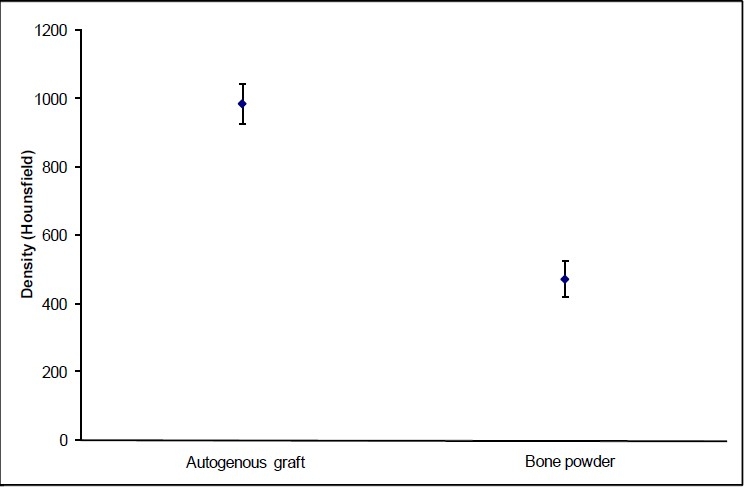
Mean bone densities obtained from cortical bone graft and bone dust. Paired t test (*P* < 0.001)

A total of 108 burr holes were reconstructed in 23 patients surgically treated for neurological conditions. Thirty-six of these bone defects were corrected with cortical bone discs (33.3%), and the remaining 72 with bone dust (66.6%), both autogenous. Most patients were women (87%). Ages ranged from 20 to 70 years, with a mean age of 45 years. Patients were surgically treated for benign tumors (30.7%), ruptured (26.1%) and unruptured aneurysms (21.7%), and arteriovenous malformations (21.7%). Complications included vasospasm (8.6%) and urinary tract infection (8.6%). All patients received dexamethasone for a mean period of 8.5 days. They remained hospitalized for a mean period of 12 days and were followed up for 9–25 months after surgery. Burr holes reconstructed with cortical bone graft had a mean density of 987.95 ± 186.83 HU according to QCT, whereas the mean density obtained from the areas filled with bone dust only was 473.55 ± 220.34 HU (*P* < 0.001) [Figure 2].

Each reconstructed burr hole was graded from 0 to 10 according to its cosmetic result, 12 months after surgery. The mean esthetic scores were 9.5 for bone fragment and 5.7 for bone dust (*P* < 0.001). General surgeons attributed higher scores to both groups when compared with the scores provided by plastic surgeons. This difference was not statistically significant.

## DISCUSSION

The choice between autogenous graft and alloplastic implants when performing cranioplasties has long been a matter of debate. There is a huge number of alloplastic materials available, although their use implicates higher costs and risks.[3,8,9,15,18,19] Many authors advocate the use of autologous graft for cranial reconstruction because of its lower cost, lack of immune response after implantation, and inexistence of infectious agent transmission.[1,17] Its capabilities of osteoconduction, osteoinduction and osteogenesis are maximal. In addition, the type of reconstruction described in the present study carries no risk to the donor site, since the bone plug is created from the inner table of the bone flap, with no additional cost.

There are some reports of burr holes filled in with compact bone dust showing good results.[2] However, no comparison between different types of grafts was performed, bone density was not determined, and fibrin glue was sometimes needed to fixate the grafted bone dust, increasing the costs.

Our difficulty to accurately quantify results remains as one of the major current limitations for evaluating the biological development of human bone grafts.[13] It is not ethical to remove implanted bone grafts in order to analyze their degree of integration, a fact that interferes with our capability of determining the time needed to achieve adequate fusion and the degree of graft reorganization. Prolo *et al*.[13] stated that palpation and visual inspection are not suitable for an objective evaluation, allowing only for qualitative analysis. Keyak *et al*.[7] and Levi *et al*.[11] used tomography to identify femoral bone density. More recently, tomography has been used to study orthodontic implants.[10,16]

**Figure 3:**
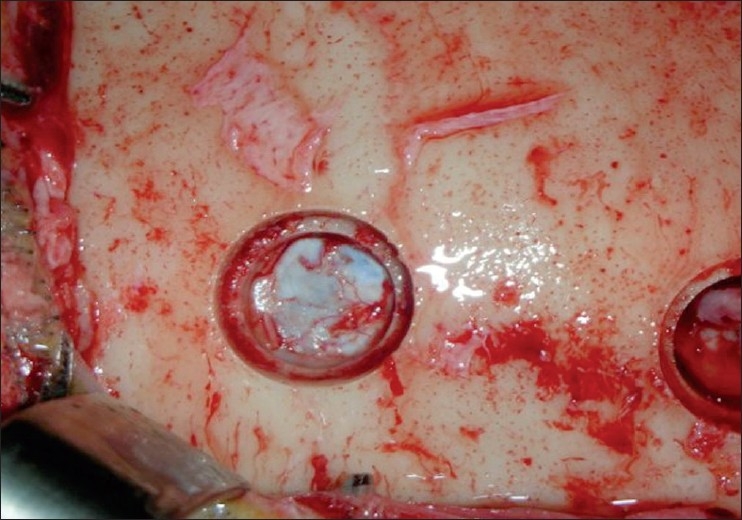
Burr hole insight

Burr holes created by neurosurgical trephines have a smaller inner diameter when compared with their outer diameter, creating a “step” over which the autograft can rest with perfect fit and there is no need for additional fixation [Figure 3]. Benefits regarding this type of autograft include no additional cutaneous incision, no need to use synthetic material or cadaveric tissue, and consequently no risk of foreign body reaction. In contrast to this, other methods of reconstruction usually require specific materials for fixating the graft, increasing the costs.[1,3–6,12,14]

**Figure 4:**
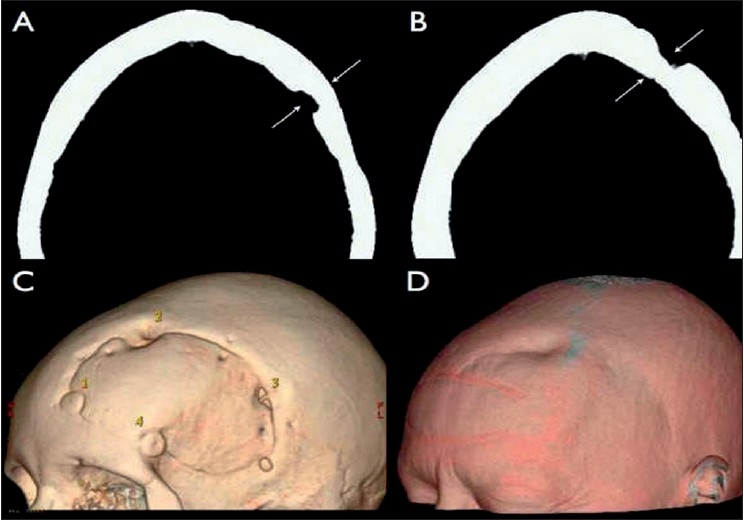
A and B show, respectively, axial tomographic views of burr holes #1 and #2, three-dimensionally demonstrated in C. Burr hole #1 was reconstructed using autogenous cortical bone graft, which resulted in good alignment with the surrounding external table (A) and, consequently, gave adequate cosmetic result (C and D). On the other hand, burr hole #2 was treated with autogenous bone powder, and the control image shows a residual bone defect (B and C), which remains visible through the skin (D)

After a variable period of time, cranial defects treated only with bone dust demonstrate local surface depressions probably caused by a high level of bone resorption, which leads to unfavorable cosmetic results [Figure 4]. Lower scores were attributed to both types of reconstructions by plastic surgeons, probably reflecting their higher concern with esthetical aspects when compared to general surgeons.

These analyses demonstrate the superiority of cortical bone graft for cranial burr hole reconstruction when compared with bone dust because of its significantly better density, which leads to better cosmetic results.

## CONCLUSION

The use of autologous bone discs obtained from the inner cortical table showed better results than bone dust for the reconstruction of cranial burr holes because of their lower degree of bone resorption and, consequently, better cosmetic results. The lack of donor site morbidity associated with low cost qualifies the cortical autograft as the first choice for correcting cranial defects resulted from neurosurgical trephines.
